# Pyridostigmine Mitigates Methotrexate-Induced Liver Fibrosis in Rats: Association with Changes in BMP-9, SIRT1, and Endoglin Expression

**DOI:** 10.3390/biomedicines13061502

**Published:** 2025-06-19

**Authors:** Mehmet Ulusan, Mumin Alper Erdogan, Ozkan Simsek, Hilal Ustundag, Zafer Dogan, Bertug Bekir Ciftci, Mesih Kocamuftuoglu, Imdat Orhan, Oytun Erbas

**Affiliations:** 1 Department of Internal Medicine, Faculty of Veterinary Medicine, Burdur Mehmet Akif Ersoy University, Burdur 15030, Turkey; mehmet.bucak@hotmail.com; 2 Department of Physiology, Faculty of Medicine, Izmir Katip Celebi University, Izmir 35620, Turkey; alpero86@gmail.com; 3 Department of Physiology, Faculty of Veterinary Medicine, Burdur Mehmet Akif Ersoy University, Burdur 15030, Turkey; profdrsimsek@gmail.com; 4 Department of Physiology, Faculty of Medicine, Erzincan Binali Yıldırım University, Erzincan 24100, Turkey; 5 Department of Surgery, Faculty of Veterinary Medicine, Tekirdag Namik Kemal University, Tekirdag 59010, Turkey; zdogan@nku.edu.tr; 6 Department of Veterinary Surgery, Health Science Institute, Erciyes University, Kayseri 38280, Turkey; 4022740002@erciyes.edu.tr; 7 Department of Obstetrics and Gynecology, Faculty of Veterinary Medicine, Burdur Mehmet Akif Ersoy University, Burdur 15030, Turkey; mkocamuftuoglu@mehmetakif.edu.tr; 8 Department of Anatomy, Faculty of Veterinary Medicine, Erciyes University, Kayseri 38280, Turkey; imdatorhan@erciyes.edu.tr; 9 Faculty of Medicine, Biruni Research Center (BAMER), Biruni University, Istanbul 34015, Turkey; oytunerbas@yahoo.com

**Keywords:** methotrexate, pyridostigmine, liver injury, fibrosis, oxidative stress, sirtuin 1

## Abstract

**Background and Objectives:** Methotrexate (MTX) is a widely utilised pharmaceutical agent in the treatment of various malignancies and inflammatory diseases. However, its clinical utility is often constrained by its potential for hepatotoxicity. Although pyridostigmine is a well-established reversible acetylcholinesterase inhibitor, its potential therapeutic role in preventing hepatic injury remains incompletely defined. The present study aimed to investigate whether pyridostigmine provides protective effects against MTX-triggered liver damage in a rat model. **Methods:** Thirty-six female Wistar albino rats randomly assigned to three groups: control (*n* = 12), MTX + saline (*n* = 12), and MTX + pyridostigmine (*n* = 12). Hepatotoxicity was induced by a single-dose MTX injection (20 mg/kg), followed by daily oral administration of either pyridostigmine (5 mg/kg) or saline for ten consecutive days. Hepatic function markers, oxidative stress parameters, fibrosis-associated mediators, and histopathological changes were assessed. **Results:** Pyridostigmine significantly attenuated MTX-induced elevations in plasma alanine aminotransferase (*p* < 0.05) and cytokeratin-18 levels (*p* < 0.001), and reduced liver and plasma malondialdehyde (MDA) levels (*p* < 0.05). Additionally, pyridostigmine treatment resulted in reduced levels of transforming growth factor-beta (*p* < 0.05), bone morphogenetic protein-9 (*p* < 0.001), and endoglin levels (*p* < 0.05), as well as increased sirtuin 1 level (*p* < 0.05). Histopathological examination revealed that pyridostigmine treatment significantly reduced MTX-induced hepatocyte necrosis, fibrosis, and cellular infiltration. **Conclusions:** Pyridostigmine exerted hepatoprotective effects against MTX-induced liver injury by attenuating oxidative stress, restoring SIRT1 expression, and suppressing pro-fibrotic signaling. These findings indicate that pyridostigmine may hold therapeutic potential for the prevention of MTX-associated hepatotoxicity.

## 1. Introduction

Methotrexate (MTX), an antimetabolite and antifolate agent, is commonly prescribed in the treatment of various malignancies (such as breast, lung, and skin cancers), autoimmune diseases, and inflammatory disorders including rheumatoid arthritis, psoriasis, and inflammatory bowel disease [1,2]. However, its long-term use is frequently limited by hepatotoxicity, necessitating regular monitoring of liver function and, in severe cases, liver biopsies [3].

The pathophysiology of MTX-induced hepatotoxicity is multifactorial, involving oxidative stress, mitochondrial dysfunction, inflammatory cytokine production, and activation of fibrogenic signaling pathways [4,5]. MTX disrupts several metabolic processes, including folate metabolism and single-carbon transfer reactions, increases homocysteine levels, and promotes the generation of reactive oxygen species (ROS), leading to lipid peroxidation and cellular damage [6]. Furthermore, a central feature of MTX-induced hepatic fibrosis is the activation of hepatic stellate cells and excessive deposition of extracellular matrix components, largely driven by transforming growth factor-beta (TGF-β), a key profibrotic cytokine [7,8]. Members of the TGF-β superfamily, such as bone morphogenetic protein-9 (BMP-9) and its co-receptor endoglin, also contribute to hepatic fibrogenesis by promoting stellate cell activation and matrix accumulation [9,10]. In contrast, sirtuin 1 (SIRT1), a NAD+-dependent deacetylase, has emerged as a crucial negative regulator of liver fibrosis. SIRT1 exerts hepatoprotective effects through multiple mechanisms, including the attenuation of oxidative stress, inhibition of pro-inflammatory signaling pathways, and regulation of cellular metabolic homeostasis [11,12,13,14].

Recent evidence suggests that liver fibrosis, traditionally considered irreversible, may undergo regression following removal of the injurious stimulus and restoration of the balance between fibrogenesis and fibrolysis [8,11]. This paradigm shift has led to growing interest in the development of pharmacological agents with antifibrotic potential, particularly in the context of drug-induced liver injuries such as those caused by MTX. Given the multifactorial nature of MTX-induced liver injury, which involves both oxidative stress and fibrogenic pathways, increasing attention has been directed toward identifying therapeutic agents capable of simultaneously targeting these pathological mechanisms. Agents with combined antioxidant, anti-inflammatory, and antifibrotic properties may offer enhanced protection against MTX-induced hepatotoxicity.

Pyridostigmine, a carbamate-derived cholinesterase inhibitor with reversible action, is clinically employed to manage the symptoms of myasthenia gravis and as a pre-exposure prophylactic against organophosphate poisoning [15]. In addition to its classical cholinergic effects, accumulating evidence suggests that pyridostigmine may exert anti-inflammatory and antioxidant effects through modulation of the cholinergic anti-inflammatory pathway and enhancement of vagal tone [16,17]. Activation of this pathway reduces pro-inflammatory cytokine release and attenuates oxidative stress [18,19,20], mechanisms that may be particularly relevant in the context of drug-induced liver injury. However, the potential hepatoprotective effects of pyridostigmine in MTX-induced hepatotoxicity remain largely unexplored.

Therefore, the present study aimed to investigate the potential protective effects of pyridostigmine on MTX-induced liver injury in rats. We specifically evaluated markers of oxidative stress, such as malondialdehyde (MDA) as well as markers of hepatocellular damage (ALT and cytokeratin-18), and fibrosis-related factors (TGF-β, BMP-9, endoglin, and SIRT1), to assess whether pyridostigmine could modulate the key pathogenic pathways involved in MTX-induced hepatic injury.

## 2. Materials and Methods

### 2.1. Animals

The experimental protocol involved thirty-six mature female Wistar albino rats (10–12 weeks old; body mass: 150–200 g) obtained from the Science University Experimental Animal Facility. Animals were maintained in stainless steel cages (two per cage) under controlled environmental parameters (temperature: 22 ± 2 °C; photoperiod: alternating 12-h light/dark) with ad libitum access to drinking water and standard laboratory chow (composition: 35% lipids, 18% proteins, and 47% carbohydrates). All experimental procedures were conducted in accordance with the Guide for the Care and Use of Laboratory Animals published by the United States National Institutes of Health and received formal ethical approval from Science University’s Animal Research Ethics Board (Approval No: 325013123, 6 November 2023).

### 2.2. Experimental Design

The animals were randomly assigned into three groups. The control group received daily oral administration of tap water (1 mL/kg) for 10 days without MTX exposure. The MTX + saline group received a single intraperitoneal injection of MTX (20 mg/kg) [21] to induce liver injury, followed by daily oral tap water (1 mL/kg) for 10 days. The MTX + pyridostigmine group received the same methotrexate protocol, followed by daily oral pyridostigmine (5 mg/kg/day) [22,23] for 10 days. The MTX dosage (20 mg/kg, i.p.) was selected based on prior studies that consistently induced reproducible hepatotoxicity in rats [21,24]. The pyridostigmine dosage (5 mg/kg/day) was chosen according to previous reports demonstrating its antioxidant and anti-inflammatory properties in rodent models [23,25], with the goal of achieving hepatoprotective efficacy while minimizing potential adverse effects.

On day 11, the rats were anesthetized with intraperitoneal ketamine (100 mg/kg, Ketasol, Richterpharma AG, Wels, Austria) and xylazine (50 mg/kg, Rompun, Bayer, Leverkusen, Germany). Blood samples were collected via cardiac puncture for biochemical analysis. After the blood collection, humane euthanasia was implemented through cervical dislocation, and surgical extraction of hepatic tissue samples was conducted for comprehensive histomorphological examination and quantitative biochemical assays. Liver fibrosis was assessed by measuring the profibrotic markers TGF-β, BMP-9, and endoglin. Hepatocellular injury was evaluated using plasma ALT, cytokeratin-18, and MDA levels as biomarkers.

### 2.3. Histopathological Evaluation

Liver tissue samples were fixed in neutral buffered formalin (10%) for 24 h, followed by dehydration in graded alcohols, clearing in xylene, and embedding in paraffin. Sections of 4 μm thickness were prepared using a rotary microtome and subjected to standard hematoxylin and eosin (H&E) staining protocols. Microscopic examination and imaging were performed using an Olympus BX51 light microscope (Olympus Co., Tokyo, Japan) equipped with an Olympus C-5050 digital camera (Olympus Co., Tokyo, Japan).

Histopathological changes in liver tissue were evaluated semi-quantitatively using Lobenhofer et al.’s scoring system. Hepatocyte necrosis, fibrosis, and inflammatory cell infiltration were graded on a scale from 1 to 4 based on the extent and severity of the observed changes [26]. Grade 1 indicated minimal changes affecting less than 25% of the examined tissue; Grade 2 reflected mild involvement of 25% to 50% of the tissue; Grade 3 corresponded to moderate changes involving 51% to 75% of the tissue; and Grade 4 represented marked pathological changes affecting more than 75% of the tissue area. All evaluations were performed by a pathologist blinded to the experimental groups.

### 2.4. Biochemical Analysis

Liver tissues were homogenized in ice-cold phosphate-buffered saline (PBS, pH 7.4) using a glass homogenizer (5 mL buffer per gram of tissue). The homogenates were centrifuged at 5000× *g* for 15 min at 4 °C, and supernatants were collected for biochemical analysis. Total protein concentration in the liver homogenates was determined using Bradford’s method with bovine serum albumin as the standard [27].

### 2.5. Determination of Liver TGF-Beta, SIRT1, BMP-9, and Endoglin Levels

The concentrations of TGF-β, SIRT1, BMP-9 (both the active and inactive form), and endoglin in liver tissue supernatants were measured using commercially available rat enzyme-linked immunosorbent assay (ELISA) kits (Biosciences, Seattle, WA, USA), according to the manufacturer’s instructions. All samples were analyzed in duplicate. Optical density values were quantified using a MultiscanGo microplate spectrophotometer (Thermo Fisher Scientific Inc., Portsmouth, NH, USA) at wavelengths specified in the respective assay protocols.

### 2.6. Determination of Lipid Peroxidation

Tissue levels of MDA, as a marker of lipid peroxidation, were quantified in liver homogenates using the thiobarbituric acid reactive substances (TBARS) method, as previously documented [21]. The procedure involved combining tissue samples with trichloroacetic acid (10% *w*/*v*) and thiobarbituric acid (0.67% *w*/*v*), followed by incubation at 100 °C for 60 min, followed by cooling on ice and centrifugation at 3000 rpm for 20 min. The absorbance of the supernatant was measured to calculate MDA concentration.

### 2.7. Determination of Plasma ALT and Cytokeratin-18 Levels

Quantification of plasma ALT and cytokeratin-18 concentrations was performed utilizing commercial ELISA test kits (USCN, Life Science Inc., Wuhan, China) with strict adherence to the protocols provided by the manufacturer.

### 2.8. Statistical Analysis

All statistical analyses were performed using SPSS software version 15.0 for Windows (SPSS Inc., Chicago, IL, USA). Prior to conducting group comparisons, the assumption of normality for continuous variables was assessed using the Kolmogorov–Smirnov test, and the homogeneity of variances was verified using the Bartlett test. Variables that satisfied both assumptions (*p* > 0.05) were analyzed using one-way analysis of variance (ANOVA) to determine statistically significant differences among the three experimental groups. For post hoc pairwise comparisons, the Tukey Honestly Significant Difference (HSD) test was employed to control for Type I errors in multiple comparisons. The data are presented as the mean ± standard error of the mean (SEM). A *p*-value of less than 0.05 was considered to be statistically significant, and *p* < 0.001 was interpreted as being highly significant.

For statistical analysis of histopathological scores, group means (*n* = 12 per group) were calculated and compared using non-parametric methods. The Kruskal–Wallis test was applied for overall group comparisons, followed by the Mann–Whitney U test for pairwise post hoc analyses, due to the ordinal nature of the scoring system.

## 3. Results

### 3.1. Effect of Pyridostigmine on Liver Fibrosis Markers

MTX administration significantly elevated hepatic TGF-β levels compared to controls (*p* < 0.01). Pyridostigmine treatment effectively attenuated this increase (*p* < 0.05 vs. MTX + saline group). Similarly, BMP-9 levels were markedly increased in the MTX + saline group (*p* < 0.001 vs. control), whereas pyridostigmine administration significantly reduced BMP-9 levels (*p* < 0.001 vs. MTX + saline group). Endoglin levels also increased following MTX exposure but were significantly decreased by pyridostigmine treatment (*p* < 0.05 vs. MTX + saline group) (Table 1).

### 3.2. Effect of Pyridostigmine on Oxidative Stress Markers

MTX administration resulted in a significant rise in liver MDA levels (*p* < 0.001 vs. control), indicating enhanced lipid peroxidation. Pyridostigmine treatment significantly attenuated MDA levels (*p* < 0.05 vs. MTX + saline group) (Table 1).

### 3.3. Effect of Pyridostigmine on SIRT1 Levels

Hepatic SIRT1 expression was markedly suppressed in the MTX-treated group compared to controls (*p* < 0.01). Pyridostigmine co-administration significantly restored SIRT1 levels (*p* < 0.05 vs. MTX + saline groups), suggesting a modulatory role of pyridostigmine in promoting SIRT1-mediated hepatoprotection (Table 1).

### 3.4. Effect of Pyridostigmine on Liver Injury Markers

Plasma ALT levels were significantly elevated in the MTX + saline group relative to the control group (*p* < 0.01), while pyridostigmine treatment significantly reduced ALT levels (*p* < 0.05 vs. MTX + saline group). Plasma cytokeratin-18 levels were also significantly increased following MTX administration (*p* < 0.001 vs. control) but were markedly decreased after pyridostigmine treatment (*p* < 0.001 vs. MTX + saline group) (Table 1).

### 3.5. Effect of Pyridostigmine on Liver Histopathology

**Figure 1:**
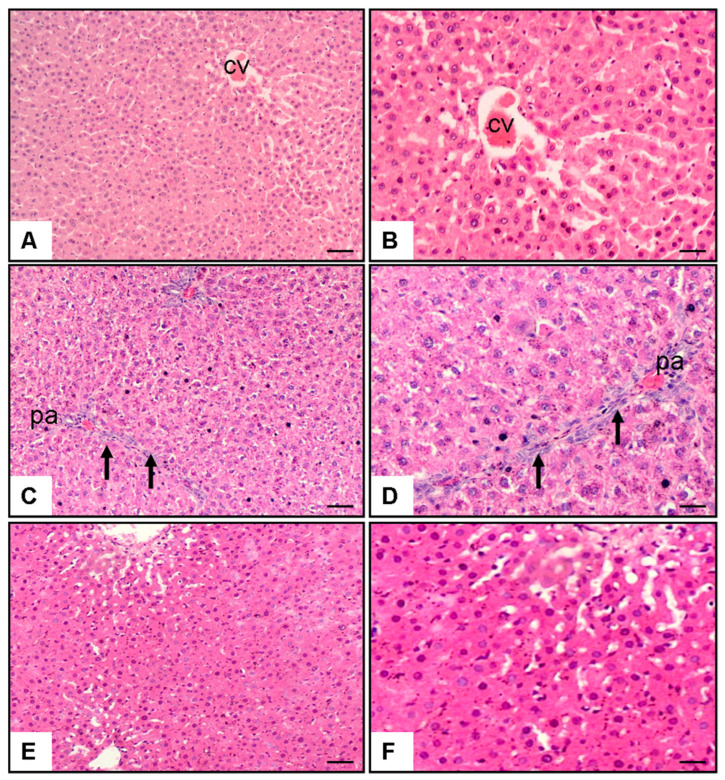
Representative liver histopathology images stained with hematoxylin and eosin (H&E) at 10× and 20× magnification. (**A**,**B**) Control group showing preserved hepatic architecture with normal hepatocytes and central vein (CV). (**C**,**D**) Methotrexate (MTX) + saline group displaying marked pathological alterations, including bridging necrosis, periportal fibrosis, and dense inflammatory cell infiltration in the portal area (pa) (arrows). (**E**,**F**) MTX + pyridostigmine group showing partial preservation of hepatic architecture with reduced necrosis, mild periportal fibrosis, and less prominent inflammatory infiltration. Scale bar: 100 µm.

Microscopic analysis of hepatic specimens revealed distinctive morphological variations among the groups (Figure 1). Control group sections (Figure 1A,B) displayed preserved hepatic architecture characterized by undamaged parenchymal cells organized in characteristic strand-like formations extending outward from the centrally positioned venous structure (CV). Hepatocytes showed normal morphology with well-defined cell boundaries, centrally located nuclei, and eosinophilic cytoplasm. Sinusoidal spaces were normal, and there was minimal evidence of necrosis, fibrosis, or cellular infiltration. In contrast, liver sections from the MTX + saline group (Figure 1C,D) showed marked histological alterations. These included extensive periportal areas (pa) with significant hepatocyte necrosis (*p* < 0.001 vs. control), characterized by pyknotic nuclei and cytoplasmic eosinophilia. Prominent inflammatory cell infiltration (*p* < 0.001 vs. control) was observed, primarily consisting of lymphocytes and neutrophils (arrows). Additionally, there was evidence of fibrosis (*p* < 0.001 vs. control), particularly in periportal regions, with disruption of the normal lobular architecture. Hepatocytes exhibited signs of ballooning degeneration, and sinusoidal dilatation was apparent in some areas. Treatment with pyridostigmine (Figure 1E,F) substantially ameliorated these MTX-induced histopathological changes. Liver sections from the MTX + pyridostigmine group showed a marked reduction in hepatocyte necrosis (*p* < 0.001 vs. MTX + saline group), fibrosis (*p* < 0.001 vs. MTX + saline group), and cellular infiltration (*p* < 0.05 vs. MTX + saline group). The lobular architecture was largely preserved, with hepatocytes displaying near-normal morphology. Sinusoidal congestion and ballooning degeneration were minimal, and residual inflammatory infiltrates were substantially decreased compared to the MTX + saline group (Table 1, Figure 1).

## 4. Discussion

The present study demonstrates that administration of pyridostigmine significantly attenuates MTX-induced liver injury in rats. The protective effects were evidenced by improvements in biochemical markers of hepatocellular injury, oxidative stress, and fibrosis, as well as by histopathological findings. To our knowledge, this is the first study to investigate the potential hepatoprotective effects of pyridostigmine in the setting of MTX-induced hepatotoxicity.

MTX-induced hepatotoxicity remains a major clinical concern, particularly for patients undergoing long-term treatment [28]. In the current study, MTX administration resulted in elevations in both plasma ALT and cytokeratin-18 levels, consistent with previous reports [29,30,31]. Increased ALT reflects hepatocellular injury, while elevated cytokeratin-18, a marker of hepatocyte apoptosis, indicates enhanced cell death [32]. Pyridostigmine treatment markedly reduced both markers, suggesting its ability to preserve hepatocyte integrity.

Oxidative stress plays a central role in drug-induced hepatic injury, including MTX-induced hepatotoxicity [33,34]. MTX exposure significantly increased MDA concentrations in both hepatic tissue and plasma, indicating enhanced lipid peroxidation. This is consistent with previous evidence showing that MTX impairs antioxidant defense systems, reduces glutathione levels, and promotes excessive generation of ROS. Ali et al. [35] reported that MTX suppresses hepatic antioxidant enzymes such as glutathione peroxidase, while Alfwuaires et al. [36] demonstrated elevated hepatic ROS, nitric oxide, and MDA levels along with diminished antioxidant capacity following MTX administration. In our study, pyridostigmine administration significantly reduced MDA levels, suggesting that its antioxidant effects contribute to hepatoprotection. These findings are consistent with those of Xue et al. [37], who reported that pyridostigmine alleviates oxidative damage in dietary fat-induced liver injury by reducing oxidative markers and enhancing antioxidant enzyme activity. The antioxidant effects of pyridostigmine are thought to involve its cholinergic properties, including activation of α7nAChR signaling and the Nrf2 pathway [37,38].

SIRT1 activation has been shown to protect against liver injury and fibrosis via multiple mechanisms, including suppression of oxidative stress, inhibition of pro-inflammatory signaling, and regulation of metabolic homeostasis [12,39,40,41]. In the present study, MTX administration significantly suppressed hepatic SIRT1 levels, in agreement with previous findings by Ilhan et al. [42]. Pyridostigmine treatment partially restored SIRT1 expression. By restoring SIRT1 activity, pyridostigmine may enhance antioxidant defenses, reduce inflammatory responses, and prevent hepatocyte apoptosis, thereby contributing to its hepatoprotective effects against MTX-induced liver injury.

The antifibrotic effects of pyridostigmine were further supported by reductions in profibrotic markers, including TGF-β, BMP-9, and endoglin. TGF-β is a key molecular mediator in hepatic fibrosis, driving hepatic stellate cell activation and extracellular matrix deposition [43]. The observed decrease in TGF-β levels following pyridostigmine treatment suggests that modulation of TGF-β signaling may contribute to its antifibrotic effect. Consistent with this, Taskin et al. [21] demonstrated that MTX elevates plasma TGF-β levels in experimental hepatotoxicity, and interventions that reduce TGF-β attenuate both liver injury and fibrosis. Additionally, BMP-9 and its co-receptor endoglin—both members of the TGF-β superfamily—have been implicated in liver fibrogenesis. Elevated BMP-9 and endoglin levels are associated with MTX resistance and enhanced fibrogenic activity [10,44,45]. In our study, the reductions in BMP-9 and endoglin levels observed following pyridostigmine treatment may represent additional mechanisms contributing to its antifibrotic effects. Collectively, these results suggest that pyridostigmine’s hepatoprotective effects may involve modulation of multiple fibrogenic pathways, including TGF-β, BMP-9, and endoglin signaling.

Histopathological findings provided additional evidence supporting the hepatoprotective effects of pyridostigmine. MTX-treated rats exhibited extensive hepatocellular necrosis, fibrosis, and inflammatory cell infiltration, consistent with previous studies [21,46]. Pyridostigmine administration significantly ameliorated these histopathological changes, preserving liver architecture and reducing necrosis, fibrosis, and inflammation. These morphological improvements were in concordance with the biochemical findings, further strengthening the evidence for pyridostigmine’s protective effects.

The protective mechanisms of pyridostigmine may involve multiple pathways. As an acetylcholinesterase inhibitor, pyridostigmine increases acetylcholine availability and enhances cholinergic neurotransmission [47]. Experimental evidence has demonstrated that the cholinergic anti-inflammatory pathway effectively suppresses inflammatory cytokine production and reduces tissue damage across various models [48,49]. Furthermore, cholinergic signaling is implicated in modulating oxidative stress and promoting cell survival [38]. In line with these mechanisms, our data demonstrated that pyridostigmine reduced oxidative stress markers, restored SIRT1 expression, and attenuated TGF-β, BMP-9, and endoglin levels, which collectively may contribute to its hepatoprotective effects against MTX-induced liver injury.

It is important to acknowledge the limitations of the present study. First, only a single dose of pyridostigmine was examined; thus, the dose–response relationship remains to be explored in future studies. Second, ELISA data revealed significant alterations in BMP-9, SIRT1, and endoglin levels. However, the absence of complementary molecular analyses (e.g., quantitative PCR for BMP-9, ALK1, ALK2, TNFα, IL-10, and IL-1β gene expression; Western blotting for phospho-Smad1/5/8; and pathway inhibition experiments) limits definitive mechanistic interpretation of pyridostigmine’s effects. Third, the current study evaluated the effects of pyridostigmine in a model of acute MTX-induced liver injury, whereas its potential benefits in chronic MTX exposure remain to be determined. Finally, pyridostigmine’s systemic cholinergic effects, including its influence on autonomic regulation and inflammatory responses in other organs, may have contributed to the observed hepatoprotective effects and present potential confounding factors when interpreting liver-specific outcomes. An additional limitation is that photomicrographic images provide only qualitative visual representations and may not fully capture minimal histopathological changes. However, such subtle alterations were detected and quantified through semi-quantitative scoring by a blinded pathologist, ensuring objective assessment of fibrosis and inflammation severity.

## 5. Conclusions

In conclusion, our study demonstrated that pyridostigmine exerts significant hepatoprotective effects against MTX-induced liver injury, as reflected by improvements in hepatocellular damage markers (ALT, cytokeratin-18), reductions in oxidative stress, partial restoration of hepatic SIRT1 expression, and suppression of fibrosis-related mediators (TGF-β, BMP-9, and endoglin). These protective effects were further supported by histopathological findings showing reduced necrosis, fibrosis, and inflammatory infiltration. While the precise mechanisms remain to be fully elucidated, pyridostigmine’s ability to increase acetylcholine availability via acetylcholinesterase inhibition may activate cholinergic anti-inflammatory pathways, contributing to its observed effects. Importantly, the present findings demonstrate associations rather than definitive mechanistic causality and should be interpreted within the context of the study’s limitations. Further molecular investigations and translational studies are warranted to clarify the underlying mechanisms and to evaluate the therapeutic potential of pyridostigmine in clinical settings involving MTX-induced hepatotoxicity.

## Figures and Tables

**Table 1:** Biochemical and histopathological parameters in experimental groups (*n* = 12).

	Control	MTX + Saline	MTX + Pyridostigmine
Liver TGF-β level (pg/g)	0.45 ± 0.1	2.1 ± 0.2 *	1.6 ± 0.3 ^#^
Liver MDA level (nmol/g tissue)	27.5 ± 0.3	58.1 ± 2.7 **	39.7 ± 0.6 ^#^
Liver SIRT1 level (pg/mg)	2.16 ± 0.1	0.95 ± 0.08 *	1.33 ± 0.1 ^#^
Liver BMP-9 level (pg/mg)	0.62 ± 0.2	1.5 ± 0.1 **	0.8 ± 0.1 ^##^
Liver Endoglin level (pg/mg)	0.84 ± 0.08	1.95 ± 0.1	1.12 ± 0.05 ^#^
Plasma Cytokeratin-18 level (ng/mL)	0.9 ± 0.2	2.62 ± 0.4 **	1.1 ± 0.2 ^##^
Plasma MDA level (nM)	28.2 ± 0.6	136.1 ± 5.3 **	91.5 ± 2.2 ^#^
Plasma ALT (IU/L)	19.1 ± 0.4	38.7 ± 0.9 *	27.1 ± 0.5 ^#^
Hepatocyte necrosis	0.1 ± 0.1	1.9 ± 0.2 **	0.5 ± 0.1 ^##^
Fibrosis	0.1 ± 0.1	1.7 ± 0.1 **	0.6 ± 0.1 ^##^
Cellular infiltration	0.1 ± 0.1	1.1 ± 0.2 **	0.7 ± 0.1 ^#^

Results were presented as mean ± SEM. Statistical analyses were performed by one-way ANOVA. * *p* < 0.01, ** *p* < 0.001 different from control groups; ^#^ *p* < 0.05, ^##^ *p* < 0.001 different from Methotrexate (MTX) + saline group. TGF-β, transforming growth factor-beta; MDA, malondialdehyde; SIRT1, sirtuin 1; BMP-9, bone morphogenetic protein-9; ALT, alanine aminotransferase.

## Data Availability

All data obtained from this study are included in this article.
